# Awareness, Policy, Privacy, and More: Post-Secondary Students Voice Their Solutions to Cyberbullying

**DOI:** 10.3390/ejihpe10030058

**Published:** 2020-08-20

**Authors:** Chantal Faucher, Wanda Cassidy, Margaret Jackson

**Affiliations:** 1 Centre for Education, Law, and Society, Simon Fraser University, Burnaby, BC V5A 1S6, Canada; 2 Faculty of Education, Simon Fraser University, Burnaby, BC V5A 1S6, Canada; cassidy@sfu.ca; 3 School of Criminology, Simon Fraser University, Burnaby, BC V5A 1S6, Canada; margarej@sfu.ca

**Keywords:** cyberbullying, post-secondary, solutions, education, awareness, privacy, anonymity, policy, campus culture

## Abstract

This paper discusses solutions to cyberbullying posed by post-secondary students from four Canadian universities. The qualitative data used in this analysis were drawn from one open-ended question on an online student survey completed by 1458 undergraduate students, as well as 10 focus group transcripts involving a total of 36 students. Seven key themes emerged: awareness and education; policy; protecting one’s privacy; technology-based solutions; empowering better choices and responses; university culture; and disciplinary measures. The findings show that post-secondary institutions need to make preventing and curtailing cyberbullying more of a priority within their campus communities, including engaging in responsive consultation with key stakeholder groups, such as students, to develop meaningful solutions.

## 1. Introduction and Literature Review

While the issue of cyberbullying continues to rear its ugly head and new forms of cyberbullying proliferate, the quest for solutions or best practices for addressing this problem is evolving at a much slower pace. Educational institutions at all levels recognize that the issue exists and that it impacts the learning environment. Their role in addressing the problem, however, has been approached with reluctance, uncertainty, and mitigation. Post-secondary institutions, in particular, lack certainty about the role they should play, lack clarity regarding the extent and seriousness of the problem, and consequently, lack decisive action on the issue [1,2,3,4].

Understanding young people’s perspectives on cyberbullying has been recognized as a key piece in circumscribing the issue and arriving at meaningful solutions [5,6,7,8]. This paper adopts the view that post-secondary students’ voices must be heard in order to generate solutions that will have grassroots buy-in from key campus constituents and that will be considered relevant by those impacted [9,10]. Further, as Tierney and Clemens [10] pointed out: “qualitative work provides a voice and a face to those individuals whom researchers study; of note, qualitative research has the potential to provide social urgency to issues that are always voluntary” (p. 67).

The purpose of this paper is to acknowledge the voices of students as a key stakeholder group and to examine their views on solutions to cyberbullying. For this reason, the research is based on a qualitative analysis of data obtained from student surveys and focus groups. Rather than applying a particular theoretical lens, as we have done in earlier analyses of these data [11,12], we used the grounded theory perspective [13]. Such an approach allows for themes to emerge inductively from the data without necessarily being constructed through a specific theoretical interpretation.

The findings reported in this paper are based on qualitative data obtained from 1458 online student surveys as well as 10 focus groups involving 36 students from four Canadian universities. Although data were drawn from Canadian universities, it is clear that bullying and cyberbullying in post-secondary institutions have been recognized as problems around the world [1,3,14]. Internationally, researchers have endeavoured to delineate the nature, prevalence, and impacts of these issues, while also acknowledging the particular context of post-secondary institutions including policy and legal frameworks [15,16,17,18,19,20]. Most researchers have solicited input from students with regard to the nature, prevalence and impacts of cyberbullying (see, for example, [15,16,17,19,20,21,22,23,24,25,26,27,28,29,30,31,32]). Further, several of these studies have adopted a qualitative approach in order to obtain a more in-depth understanding of student perspectives [16,20,21,25]. It is, therefore, the authors’ view that student input into solutions is also required for meaningful solutions to emerge.

Dennehy and colleagues [6] conducted a systematic review of 79 qualitative research studies involving 753 youths aged 10 to 19, exploring their conceptualizations of cyberbullying, including intent to harm, repetition, accessibility of targets, anonymity of perpetrators, and barriers to disclosure. Their findings from these studies of adolescents challenge some of the commonly assumed characteristics of cyberbullying as an offshoot of traditional bullying. For instance, the traditional definition of bullying, that most researchers borrow from Olweus [33], suggests that acts defined as bullying must include an intent to harm. Research participants in the studies reviewed by Dennehy et al. [6], however, suggested that victims’ perception of harm, though subjective, is also important to consider. Postings that start out as jokes, without an intent to harm, may escalate into cyberbullying due to ambiguity or an absence of body language allowing for (mis)interpretation of intent or perception. Study participants also acknowledged that perpetrators of cyberbullying may deny their true intention by claiming that they were “just joking” or they may hide behind the anonymity of online interactions to deny their involvement. Even in instances where their identity is revealed, perpetrators may claim someone else used (or hacked) their account without their knowledge. Repeated hurtful actions, however, were generally deemed intentional. Visual forms of cyberbullying were perceived as especially damaging (see also [19,34]).

Evans et al. [35] conducted a study of adolescents at a video blogger convention where participants were asked about how to help the target of cyberbullying in a given vignette. Key ideas that emerged from the responses included: the target ignoring the cyberbullying or taking a break from social media; bystanders helping the target feel better and standing up for them; and social media websites and policy makers blocking images or users that are abusive.

In keeping with the idea that cyberbullying exists on a continuum from elementary, middle, and secondary schools to post-secondary and into the workplace [3,32,36,37], Crosslin and Golman’s [21] research with first- and second-year college students in Texas revealed similar themes to those highlighted by Dennehy and colleagues [6]. Students in this study of young adults also discussed the problems with anonymity and people masquerading as others. They noted that online interactions are easier to misconstrue than face-to-face interactions and that the school culture of partying results in some things being taken less seriously than they should, with 20% of respondents indicating that cyberbullying is a rite of passage. Some of the solutions proposed in this study included having cyberbullying be a topic addressed during first-year orientation or in a first-year course, residence advisors giving workshops, and the idea that more education is needed to dispel the ideas that normalize cyberbullying. The authors reported that a multi-level approach was needed that would encompass the individual, the organization, and the community, and be embedded in policy. Others have also remarked upon the major role played by anonymity in contributing to cyberbullying behaviours [38,39].

A key distinction between the handling of cyberbullying at post-secondary institutions compared to the K-12 school system is that emerging adults may feel that they can or should be able to deal with such matters on their own [29]. Moreover, university students may not know where to go for help [3,29,40]. Francisco et al. [22] reported that few college students turn to someone at the institution for help with cyberbullying, preferring instead to ask friends and family (see also [11,41]). They also found that post-secondary students may find it childish to report cyberbullying (see also [21]). In addition, a majority of university students are not aware whether a policy on cyberbullying exists at their institution, which is not surprising given that most are equally unaware of criminal laws regulating cyberbullying behaviour [3,19,41,42,43].

It is considered vital to have a clearly articulated policy in order to handle cyberbullying, harassment, and incivility in universities [3,11,19,43,44,45,46,47]. That policy needs to provide specific information and inspire confidence for a successful resolution of the problem in order for students to come forward with their concerns [29]. A study by Smith and colleagues [48] regarding the cyberbullying of faculty by students highlights the importance of policy that provides a clear outline of what constitutes cyberbullying and the consequences for engaging in it, responsiveness when issues arise, and swift and escalating disciplinary measures. They recommended that the policy be housed within accessible student and faculty handbooks and that raising awareness and training on the issue take place for all members of the campus community (see also [4,31,47,49,50,51]).

In Japan, Kanayama and Kurihara [52] reported on a range of preventative measures taken at one university in order to build a comprehensive multi-level support system for students. Following mandatory mental health screening for all incoming students, a personalized support plan is put into place, and a close relationship with medical institutions is established as needed. A peer support system was also developed to cultivate a sense of belonging among students. A curriculum was designed that included two courses aimed at preventing cyberbullying: “mobile phones and the law” (a compulsory first-year course) and “cyber police” (connected to a branch of the police designated to address cyber-crimes).

It is important to acknowledge that some of the behaviours described as cyberbullying are also considered criminal acts, depending on legislation that has been enacted in a given jurisdiction. For instance, Canada has applicable criminal legislation governing cyberbullying that takes forms such as: non-consensual distribution of intimate images, child pornography, criminal harassment, uttering threats, extortion, sexual exploitation, child luring on the internet, intimidation, obscene publications, identity theft and fraud, defamatory libel, hate speech, as well as applicable civil remedies for defamation, invasion of privacy, intentional infliction of mental suffering, and appropriation of personality [53]. In Australia, no specific “cyberbullying” offence exists, but some states and territories have laws prohibiting certain behaviours that are associated with cyberbullying, such as non-consensual distribution of visual content, unlawful threats, assault, and stalking [19]. In the United States, all 50 states have legislation mandating that K-12 schools have policies around bullying and cyberbullying, although few have the same for post-secondary institutions [54]. Thirty-nine states, however, have laws governing revenge porn.

In terms of the legal frameworks around cyberbullying, it is also important to note the protections around freedom of speech, which have been used to defend some of those accused of engaging in cyberbullying at universities, e.g., [55]. O’Connor and colleagues [54] offered a range of recommendations for developing and implementing cyberbullying policies at universities, while also being mindful of laws protecting freedom of speech. Such recommendations include: focusing on the impact of speech on the victim (rather than the content itself); focusing on prevention, including reporting procedures; not being overly broad (e.g., zero-tolerance); being specific to the issues in the particular campus community; and being grounded in law.

In the spring of 2014, the authors collaborated with colleagues on the organization of a symposium on cyberbullying at universities. The symposium was an opportunity to share some preliminary research findings on this topic and to invite dialogue among various stakeholders (post-secondary students, faculty members, university staff, and administrators/policymakers from British Columbia and surrounding areas) about the issue and solutions to cyberbullying at the university level. Notes about the issues discussed have been summarized and shared publicly [56,57]. Key themes that arose from the dialogue on solutions to this problem included: a clear and transparent policy development and implementation process; acknowledgement of the legal and cultural context; awareness raising and education; training and support for all involved; and creating a kinder culture in post-secondary institutions and beyond. For a full discussion, see [57].

Therefore, the question posed in the present research and explored in this paper is to determine students’ perspectives on the solutions to cyberbullying in post-secondary institutions. By emphasizing student voices, it is hoped that we can arrive at relevant solutions that are effective and also resonate with this key group of stakeholders.

## 2. Materials and Methods

This paper examines two sets of data pertaining to students’ suggestions for resolving cyberbullying in post-secondary education. The data used here are part of a broader study, aspects of which have been reported elsewhere [11,12,44,45,58]. Quantitative analyses of students’ views pertaining to solutions have been detailed in a separate paper [11]. The focus of this paper is a qualitative thematic analysis of students’ written and oral comments from survey and focus group data using a grounded theory approach [13]. The data were examined in close detail and in successive iterations in order to allow key themes to emerge [59,60], rather than to lend a particular interpretation based on the authors’ prior awareness of the issues drawn from their earlier work. Although the authors used their prior knowledge of cyberbullying at K-12 to shape focus group questioning protocols (see Appendix A), they were cognizant of the differences in context and age of the participants, and the possibility that quite different solutions might emerge with university students.

The first dataset was from one of the open-ended questions in the online survey of undergraduate students at four Canadian universities asking respondents: “In your opinion, what are some ways for preventing or curtailing cyberbullying that the university or students themselves should do?” Of the 1925 survey respondents, 1458 provided a response to this question. This question was posed prior to a closed-ended question that asked respondents to select their top five ranked choices from a list of 14 suggested solutions. This ordering of the questions allowed for students’ initial thoughts on solutions to be recorded prior to any suggestions being made that might influence their views. Although the survey was quite lengthy, the fact that 75.7% of respondents provided a response to the open-ended question suggests a good representation of the overall sample. The overall sample included students of different gender identities, racial and ethnic identities, academic programs, and years of study [11].

The second data source was a set of 10 transcripts from in-person focus groups conducted with student volunteers who had previously completed the survey. In total, 36 participants were involved in the focus groups, which spanned the four universities (see Appendix B for demographic information collected about focus group participants). Each focus group was led by one of the researchers and lasted between 45 and 75 min. The focus groups were semi-structured, in that participants were led through the protocol questions (see Appendix A), but these questions were purposely broad in order to provide space for discussion of the issues that participants found most relevant, allowing them to elaborate those ideas as needed. Focus groups ended once all questions had been asked and participants no longer had anything further to add when prompted with the last question of “What else would you like to tell us about the solutions to cyberbullying?”

Both the surveys and the focus groups were conducted following an approved application to the research ethics board of each participating university. All participants provided informed consent prior to being included in the study. The study was conducted in accordance with the Declaration of Helsinki, and the protocol was approved by the Office of Research Ethics at Simon Fraser University (#2012s0546 approved 31 July 2012). Online surveys were conducted anonymously, and pseudonyms were used for all focus groups. Participants were assured that the researchers would not disclose any identifying information. Participants in the online survey could have their name entered in a draw for one of five 20 dollar gift cards if they chose to do so. The draw information was collected separately from the survey data in order to preserve the anonymity of the survey data. Each participant in the focus groups received a 5 dollar gift card as a token of appreciation for their participation.

Focus groups were audio-recorded and transcribed. The transcripts were coded using NVivo software version 10.0.3 (QSR International, Burlington, MA, USA) into themes identified by the researchers based on earlier analyses of the surveys as well as new themes that arose through an iterative process of open coding [59,60,61]. The initial coding of all data was conducted by one of the three primary researchers and verified by the other two for coding consistency and consensus. The investigators agreed on additional themes to be added based on this initial interaction with the data and a second round of coding was conducted to incorporate these adjustments. A final round of coding was conducted to verify the accuracy of the coding and to ensure all themes agreed by the three researchers had been reflected. The survey question data were then examined in relation to the themes elaborated from the focus groups. No additional themes arose from the analysis of the survey data; however, certain inconsistencies in the ways in which particular themes were discussed or emphasized were noted. The consistencies found between different data collection methods, different sources of data, and different researchers generally point to the reliability of the following analysis [59,60].

## 3. Results and Discussion

The analysis of the two data sources produced several themes regarding solutions to cyberbullying. There were some differences between the responses provided anonymously in the online survey and those provided in person in the focus groups. However, since there was substantial overlap between the responses provided in both forums, and any differences were mainly in relation to emphasis or priority, the key themes that emerged from each group are discussed together in order to avoid repetition. Discrepancies between the two sets of findings are highlighted where appropriate.

Dominant responses were identified within each dataset based on the frequency and/or strength of the responses within the broad category of solutions [60,61]. The quotations identified in the following pages are illustrative of these dominant responses.

As previously noted, the survey also included a closed-ended question regarding solutions where respondents were asked to rank their top five solutions from a list of 14 options [11]. In this part of the survey, students ranked counselling, anonymous reporting, respectful culture, punitive measures, and policymaking as their top five solutions. However, in the open-ended question that preceded the rank ordering one and invited students to write in their solutions, the following five top solutions emerged: awareness/education; taking measures to protect one’s privacy; reporting and confronting such behaviours; disciplinary measures; and ignoring it. Other less frequently recurring themes worth noting were the importance of anonymity, policy, and university culture.

Focus group responses included many of the same themes identified in the open-ended survey question. The top five themes may be described as: policy; awareness/education; technological solutions (anonymity, privacy, blocking, reporting, monitoring, login pop-up warnings); empowering better choices (ignoring, bystanders’ roles, empathy and compassion); and university culture. When asked specifically “If you had the power to make one change at the university that would prevent, curtail, or stop this kind of behaviour, cyberbullying, what would that be?”, students gravitated toward policy and awareness/education measures, as well as reducing people’s ability to be anonymous online.

After carefully comparing the themes that emerged from the open-ended question on the survey and in the focus groups, the following seven themes were determined as being dominant to both groups and are labelled as: awareness and education; policy; protecting one’s privacy; technology-based solutions; empowering better choices and responses; university culture; and disciplinary measures. The list and its ordering reflect the relative emphasis placed on the different solutions as identified by participants in both parts of the study, starting with the most dominant.

### 3.1. Awareness and Education

The open-ended survey responses accentuated awareness and education above all other themes, and this solution was also emphasized emphatically in the focus groups. Many of the focus groups took up the idea that awareness raising should be started at a much earlier age than university. As Flower, a third-year student, stated:

I think the best place to start is at the very young impressionable ages […] elementary school I think, kids at that age, they are just so eager to make things better in and they have that positive outlook and trying to fix any problem that comes their way.

(Flower, University B, Focus Group 2)

Many in the focus groups were not aware of the extent of the problem at university, especially if they had not experienced or witnessed it themselves. They attributed this lack of knowledge to a failure to disseminate information about the problem, suggesting that more needs to be done to educate the university community about the nature, prevalence and impacts of cyberbullying, the policies that govern cyberbullying, and the services available for those targeted. As Justin, a second-year student, noted: “If people know the effect it has on people, I think that would greatly decrease how much [cyberbullying] is going around” (Justin, University D, Focus Group 3). Or, as Jay said:

I actually had no idea about any policy. I don’t know if that’s my fault for not like reading it, but I feel like it’s just, yeah, I didn’t. I actually didn’t know until doing the survey that we may not have a policy […] students just don’t really know, they probably don’t know who they can reach out to cause the counselling services, there’s like a little office and a sign, but I don’t know how many kids will kind of get out of their shell and seek that help.

(Jay, University B, Focus Group 1)

Students said that if the university has a policy, it must be effectively communicated to students, faculty and staff:

I think the biggest thing is to have a system to discreetly and properly deal with the situation and support the victim, and making sure people are aware of it. I for instance, am unsure who, specifically, I should contact if I ever did experience cyber-bullying through the university and what exactly would be done about it.

(Survey respondent 974, University A)

The students also had specific suggestions for increasing awareness about cyberbullying, including: first-week orientation events; course syllabi and discussion during the first class of every course; developing a broader university life skills course that included cyberbullying; specific events; and poster campaigns. Raising the issue during orientation week at the start of a school year was seen as an opportune time to “set the tone” regarding online behaviour. Outlining the problem and the expected behaviour on course syllabi, as well as reinforcing it during the beginning of a course would communicate the message to those who did not attend orientation week.

Maybe mention at the start of each semester in the first day of classes about the policies. Perhaps have students sign a waiver on the first day of classes stating they know (a) what cyber bullying is, (b) that any form of cyber bullying will not be tolerated, (c) any accusations of cyberbullying will be treated with the utmost importance and students caught will be reprimanded academically.

(Survey respondent 435, University A)

Students also said that courses with an online component absolutely should include information about the tone and tenor of online interactions, including a mandatory online tutorial as to what is acceptable and unacceptable behaviour, and regular monitoring of comments.

I think the biggest way for professors, teaching assistants, and tutor markers to help reduce cyber-bullying is to monitor the chat-rooms online for the course. They should also warn students that all comments will be reviewed and if they post inappropriate content, they will be removed from the chat-room.

(Survey respondent 438, University A)

Some students also felt that a broader university life skills course or a series of workshops in first year like those being offered at some other post-secondary institutions would be beneficial. A course of this nature might include study skills, writing skills, time management, physical and mental health, self-esteem, confidence, how to cope with stress, how to help others, and plagiarism, as well as cyberbullying. Many students, in fact, referenced the raising of awareness around plagiarism as an exemplar that could be emulated in educating post-secondary students about cyberbullying, since plagiarism is “drilled into them” from Day 1. The following exchange between two fourth-year focus group participants illustrates this parallel:

X: Yeah, it can be a little gray. What’s plagiarism? What’s not?

Julia: Yeah.

X: It can be, you can have legal implications to it or it just can have people chastising you but it is strongly reinforced that you should not do it and I think in the same way it kind of, it’s just asking people not to do it out of good faith and I think cyberbullying is kind of like that too.

Julia: Yeah, building on that, I think a lot of people don’t understand that. You know, patch writing is a form of plagiarism and everything like that but you have to like do, either your teacher explains it, makes you read the, makes you do that online tutorial, I think almost doing something like that to do with cyberbullying, because it may not be an issue to a lot of people but also, when I came into this I was like ‘oh there’s no cyberbullying’. So people can also have that mind set and maybe just having a brief orientation because it could completely impact somebody’s school experience, having that brief introduction, brief five question tutorial […], it could just overall affect your university experience, especially how technology is used in every class now.

Students also mentioned raising awareness through specific anti-cyberbullying events, although they recognized that such events are voluntary and may not reach the intended audience. As Lux, a third-year student, pointed out: “Yeah, I mean, it’s so hard as an optional thing, because the people who are interested in it will go to it. Those who probably are bullies or whatever, they’re gonna be like ‘whatever’ and not go” (Lux, University A, Focus Group 2). Students living in residence also mentioned how difficult it is to garner attendance at planned events.

Poster campaigns also can be effective as the campaigns can have some impact by repeatedly drawing attention to the issue if placed in high traffic areas. One focus group participant gave an example of a pink poster found in all the washrooms at University D that encouraged students to walk up the stairs rather than taking the elevator in order to save electricity and prevent obesity; it “just sticks with you” (Hannah, fourth-year, University D, Focus Group 1). One of the survey respondents suggested a poster campaign on campus against cyberbullying that said “pause before you post” (Survey respondent 47, University C).

In addition to specific educational programs and awareness strategies about cyberbullying, students also noted the need for education regarding some of the broader underlying attitudes that contributed to cyberbullying.

I think cyber bullying and bullying in general is an offset of multiple factors—racism, sexism, misogyny, homophobia, judgements on behaviour and appearance, and many more. I think that in order to really put a dent on bullying and cyberbullying we need to focus on educating people on multiculturalism, freedom of sexual preference, the importance of personality over appearance, and just tolerance. I understand that not all people will get along, but tolerance is important.

(Survey respondent 416, University A)

### 3.2. Policy

Focus group participants, more so than survey respondents, zeroed in on policy solutions as a priority. In fact, when they were asked: “If you had the power to make one change at the university that would prevent, curtail, or stop this kind of behaviour, cyberbullying, what would that be?”, several of the groups chose policy as their primary recommendation. In discussion, the groups recognized that policy had the potential to shape all (or most) of the other solutions. Students talked about the importance of creating a normative positive online environment that would then become embedded in university policies. They also felt that a stand-alone policy on cyberbullying, or at least specific references made to cyberbullying in other policies such as student conduct, are needed as well. As Emily stated:

I would definitely say writing rules regarding cyberbullying more definitively into the Student Code of Behaviour at the university […] you direct students to that, just like you can direct them to plagiarism, just like you can direct them to cheating and it’s right there, it’s written in plain English and they know they’re not allowed to do it.

(Emily, fourth-year, University D, Focus Group 3)

Focus group students also felt that rapid changes in technology required that policy keep up, as illustrated by Berry’s comment:

I also agree about establishing a policy just strictly against cyberbullying. I think that it’s something that needs to be adopted by many different institutions like high schools and now at post-secondary, even within our own legal system. We kind of have to incorporate a modernization of technology.

(Berry, second-year, University B, Focus Group 3)

While survey respondents did not identify policy development as the top priority, they did acknowledge its importance. Several students said that “zero tolerance” policies should be in place, while others suggested: “Strict guidelines which are widely and clearly distributed; enforcement of these guidelines; well communicated means of reporting cyber bullying” (Survey respondent 150, University D). Survey respondents also identified the need for procedures for “reporting” such behaviours, “regulating” and “monitoring” university sites, as well as a range of disciplinary measures that should be taken against perpetrators of cyberbullying. For example, one survey respondent wrote: “encourage victims to report cyber bullying to designated authority which can investigate the issue. I am not aware of any department in school that actually looks after that. There should be consequences to whomever that engages in cyber-bullying” (Survey respondent 73, University B).

But while developing and communicating cyberbullying policies and procedures are important, students in both groups noted that they also must be enforced consistently, fairly, and effectively:

Have a set list of rules and consequences that are enforced as uniformly and as fairly as possible. Take complaints and victims seriously.

(Survey respondent 109, University D)

Really promote awareness of how to end it and who to go talk to. There should be someone I can go talk to about harassment who will actually enforce rules against cyber-bullying and confront the people doing it. The Human Rights and Equity people are ABSOLUTELY useless. […] I had a situation where I was very badly bullied by a group of students at [University A], and I went to the Human Rights and Equity people, and they did ABSOLUTELY NOTHING. They just told me to avoid the people. So I did—I started taking different classes to avoid them. Why did I need to make the inconvenient change? Why couldn’t those students be disciplined? I was very, very unimpressed with these “Human Rights” people and it really discouraged me from bothering to tell anyone about issues in the future.

(Survey respondent 525, University A)

The latter quote illustrates that while it is important to have relevant policies in place, their enforcement needs to be, and to be perceived as, effective, otherwise the policies become somewhat meaningless.

### 3.3. Protecting One’s Privacy

While taking measures to protect one’s own privacy did not feature prominently in the focus groups, self-protective measures were the second most common response to solutions identified in the open-ended survey responses. In the anonymous context of the survey, a large number of students expressed the view that cyberbullying could be curtailed by securing one’s devices with passwords, not sharing passwords, not leaving devices unattended, logging out of devices and applications, placing a high degree of privacy settings on all social media accounts, being selective of those allowed into one’s social media circles, and being careful about what one posts and who can see it. Students were blunt about the need for everyone to take precautions to protect their privacy. Comments such as these were typical:

Keep privacy setting on. Don’t share personal information online. Refrain from having inappropriate pictures of your self taken (in other words, behave). Do not approve tags in pictures that you would not want an employer to see. Only allow close friends to contact you online.

(Survey respondent 388, University A)

First and foremost, they should practice safe surfing. It is a rule for me to maintain a certain anonymity online and put very few identifiers online. Only people who are close to me are allowed to contact or search for me. I do not post photos online. If you give people tools to use things against you then they will.

(Survey respondent 152, University C)

Don’t add people you do not trust completely on FB or other social networking sites, i.e., strangers, people who you are acquainted with but you are not friends with. Don’t bother even commenting on Youtube—people are very rude. Put very little information/pictures about yourself on these sites so no one can manipulate this information.

(Survey respondent 202, University B)

Some people don’t understand that the Internet will never be a safe place, and they need to know that anything shared anywhere, especially Facebook, will be kept on file and used with that person’s associated IP and name for the rest of their lives. This means that if a “cyber bully” ever targets someone who is essentially unsecure on the Internet, they are able to find any and all information to torture that victim.

(Survey respondent 29, University C)

Focus groups respondents held similar views about the need to be proactive about privacy; for example:

I think initially on an individual level just picking and choosing who you’re gonna invite into your social network, I don’t think it should be an open thing. I do believe in keeping your profile as private and kind of like how I was saying with my Twitter and only inviting particular people that you feel safe sharing information with into your social network.”

(Berry, second-year, University B, Focus Group 3)

There was some victim-blaming in the responses, see also [62,63], with some students, particularly survey respondents, saying that people too often left themselves vulnerable to being cyberbullied and therefore should not be surprised if they suffered the consequences.

To prevent cyber bullying, one must keep their information limited, be smart with passwords, and not wear their heart on their sleeve when it comes to social media.

(Survey respondent 29, University C)

A lot of people are attention seekers. When they eventually do something embarrassing, such as posting nude photos, and it blows up in their face, they are somehow the victim. Preventative measures must include letting people know that if you are sending potentially embarrassing information about yourself, it is your fault and you will have to deal with the consequences.

(Survey respondent 97, University C)

Don’t engage in other people’s business. And importantly, Have COMMON SENSE.

(Survey respondent 445, University A)

Students should be cautious in what they are commenting on. For example, if you post a status on Twitter/Facebook that is controversial and people respond negatively towards you, that’s opportunity (unfortunately) for people to target you. The same for posting promiscuous pictures of yourself on Facebook, although I wish it wasn’t this way, people can use that as an opportunity to bully you.

(Survey respondent 657, University A)

Update and/or maintain privacy settings (surprisingly not many people do so, then are upset when attacked. They shouldn’t give any stranger the opportunity first of all to try to hurt them). If they feel harassed on Facebook, delete/block the harasser.

(Survey respondent 71, University B)

In terms of students, one of the ways to prevent from being bullied is to have less information online. Such as your relationship status or your mood. In my opinion, people get cyber bullied on Facebook etc. because they post attention seeking comments on their wall. Bullies see this as an easy target to bully.

(Survey respondent 723, University A)

### 3.4. Technology-Based Solutions

#### 3.4.1. Reducing Online Anonymity

While some students lauded the benefits of anonymity on the Internet (for example self-expression), a larger number of both survey and focus group respondents identified anonymous online interactions as a factor in cyberbullying since the perpetrator can hide behind anonymity to write things they would not say in person. Students felt that if there were a way to limit anonymity, cyberbullying could be curtailed. One female student mentioned that all social media sites in her country of origin (Korea) require a social insurance number in order to log in. She felt this was much more secure and a better way to track down violators than the social media site policies in North America that require minimal identifying information, and where it is easy to provide false information (May, fourth-year, University A, Focus Group 1). Other students expressed similar concerns with anonymity:

Students feel they won’t get caught or held accountable for things over the internet. If students felt their IP address, or some other identifier would link the incidents to them, allowing them to be caught/exposed I think it would reduce the feeling of being anonymous they think they have now to do or say what they want online.

(Survey respondent 374, University B)

The internet is vast, and with its cloak of secrecy comes hollow courage. There is only a method of prevention of this cloak is taken off and burnt.

(Survey respondent 442, University A)

Empathy is removed from the equation due to that anonymity.

(Survey respondent 937, University A)

Facebook’s practice of turning a blind eye to fake profiles or fake names also increased the opportunity for perpetrators to be anonymous:

Sites like Facebook permit people to make fake profiles and people will openly slander people even from their own real profiles. Computer screens give people the ‘courage’ to say things that they would not necessarily say out loud or to peoples’ faces in real life.

(Survey respondent 152, University B)

Some students wanted universities to be more proactive and require additional login identification on their platforms or add automatically-generated pop-up messages that limit anonymity; for example: “when you log in there can be a pop-up just saying like your user name is recorded, so anything you say or just be aware, like something of your language that you’re using, this is a community space and is recorded” (Lux, female third-year, University A, Focus Group 2) and “Sites like ‘Rate my professor’ could require names which would make people less likely to give false accusations” (Survey respondent 933, University A).

#### 3.4.2. Other Technology-Based Solutions

In addition to reducing online anonymity and increasing privacy settings, students referenced other ways in which the technology could be mobilized to prevent or curtail cyberbullying, including blocking, reporting, and monitoring. Students pointed out that most social media platforms have some form of mechanism in place to block or report abuse. As one student said: “If you feel targeted by a bully, simply delete your social network account or report/block the bully” (Survey respondent 182, University C). Some students felt that universities should monitor online course discussion groups, “I think forcing people to log into groups using their Facebook is a start, and then monitoring discussion groups” (Survey respondent 261, University A).

Non-university sites that use the university’s name, such as confessions sites, “spotted at” and “overheard at” sites, and student-created sites (on Facebook, for instance) that invited students from the university to join, should also be monitored. As one student stated:

[L]ike the university being aware that there are these other pages created by students using the [university] name, accessing the [university] community that they could be monitoring and you know, just keeping an eye on it to make sure things aren’t getting too out of control […] if it’s not monitored and not checked, that’s when students start getting really brave and that’s when problems start kind of escalating.

(Emily, fourth-year, University D, Focus Group 3)

Another student from the same university pointed out that many administrators of these non-university sites on Facebook are students, and even though it is a heavy burden on them, some do monitor the sites and take down posts that are unethical (Cherry Blossom, third-year, University D, Focus Group 1). According to students, more careful attention should be given to these ways of curtailing cyberbullying.

### 3.5. Empowering Better Choices and Responses

Both survey and focus group participants talked about the need for students to learn to respond in more ethically caring and responsive ways towards each other. A number of survey respondents specifically cited the “golden rule” of treating others as we wish to be treated. Students felt that more attention should be given to building up individuals’ resilience, empathy, compassion, and respect. They acknowledged that policy and education play a role in empowering students to make better choices and help build a more respectful normative environment where negative behaviour is discouraged.

Many of the comments were focused on the need for students to build up their self-esteem, or to be strong if targeted, or to learn to ignore bad behaviour and not take it personally.

Be strong, be confident, have faith in yourself and in who you are and your own abilities and strengths. BE AWARE of your own weaknesses, do not let others’ opinions get to you, as long as you accept who you are and are also self-aware, you will be fine no matter how much bullies or cyberbullies try to attack you.

(Survey respondent 360, University B)

Like many others I would just suggest that victims should ignore the bullies and instead focus on anything that makes them happy, be that in hobbies or social interactions. There is also the common adage that the less you respond to their comments the less power you give them.

(Survey respondent 288, University B)

It’ll always happen to people, there’s honestly no way to prevent it, unless you just completely remove yourself from all social networking sites. But those who are bullied should learn to stand up for themselves and just ignore the stupid people. It’s best to just be yourself, and if other people dislike you, then whatever, that’s their issue not yours.

(Survey respondent 129, University D)

If people want to engage in such activity there is little a person or organization can do to stop them. The best thing that can be done is to help people on the receiving end to realize there will always be people who do not like them, help raise their self-esteem so that it does not affect them much, and help them learn how to minimize any damage

(Survey respondent 863, University A)

A few of the survey comments, written under the cloak of anonymity, were extremely harsh, insensitive, and punishing towards to those on the receiving end of cyberbullying, another reason for prioritizing education and awareness regarding the nature, extent, and impacts of being cyberbullied. Here are some examples:

Suck it up

(Survey respondent 399, University B)

YOU BETTER MAN THE FUCK UP

(Survey respondent 39, University B)

Have a thick skin and a good attitude

(Survey respondent 321, University A)

Lighten’ up. Don’t feed the trolls. It’s just the internet. Grow some balls.

(Survey respondent 756, University A)

University students are old enough to understand the mechanisms of blocking and ignoring people that they are getting harassed by. And if they are not, then I think it is okay that they get a little cyber bullied before realizing you can block someone or report them just so that the person getting cyberbullied will not think that everyone in the world is going to like them or be nice to them. In other words, the real world is just as unkind as the cyberworld, and you are not able to block people in the real world—grow tougher skin!

(Survey respondent 339, University A)

Students from both groups also talked about the importance of bystanders intervening when they see abusive online behaviour. Some suggested that it is often easier for a bystander to step in than it is for the target, and that sometimes a bystander’s response is more impactful. Intervening to stop the behaviour models ethical behaviour and conveys support for the target, regardless of who they are.

It’s very important in all aspects of life […] if I was to see someone I don’t know that was in need of some kind of help […] I think that that is a display of being a good citizen […]. I think it portrays a good message and sends out that that’s the kind of person you should be in a community or in a school that we all belong to and we want to work together […] that in terms of a role model, that is important.

(Berry, second-year, University B, Focus Group 3)

Making rules and fines will not help in my opinion. It needs to come from the population within itself. If someone is cyberbullying someone else, their friends need to tell them it’s wrong. Hearing it from a friend may make the cyber bully realize how wrong it is.

(Survey respondent 104, University D)

Not supporting it if you see it happening. Example: if someone is cyber-bullying someone via status or something, don’t ‘like’ it! Also, you can comment and say that’s disrespectful or something like that. Unfriending the person who is doing that so they will have less of an influence, talking to the person being bullied privately and telling them you’re sorry it’s happening to them and that it’s not true (whatever the bully is saying). You could also report the bully to someone (i.e., the police) if it’s serious.

(Survey respondent 393, University A)

Students also felt that bystanders sticking up for victims may serve as an educative function for the perpetrators as well.

[O]nce the person is confronted by someone […] just saying like hey we see you doing this, can you, like, why? And even just talking to somebody and making them aware that it’s not something they really should be doing. I think, at a university level that would be almost more effective […] because I think people generally have the sense that like once they’re confronted with something they realize like okay this is probably not right. And I think a lot of people just think it’s harmless what they’re doing, they think oh, you know, everyone does it, I can do it.

(Julia, second-year, University A, Focus Group 1)

Participants in one focus group discussed the possibility of peer-led conflict resolution or mediation, with an emphasis on fostering empathy and closure, although this process was not mentioned in other groups.

I feel like it would be so helpful for the bully to see the person who they victimized face-to-face and actually talk to them, see how they’re feeling, how they affected the person. I feel like they would come to a better realization.

(Elena, second-year, University B, Focus Group 1)

### 3.6. University Culture

Participants noted that it is a challenge to counter cyberbullying when campus culture is situated within a broader internet culture where rudeness, foul language, negativity, superficiality, and the idea of “toughening up” tend to be valued and reinforced. Students pointed to the online gaming culture as an example where participants are expected to be nasty to each other.

Educate them the dangers of anonymity and the harsh internet culture. Most young males will learn the unwelcoming world of the internet through online video games and ease into it. Females, though, must look for another way because of their lack of interest in video games. Educating them before having to realize on their own that the world through the screen is much more scary than anything else.

(Survey respondent 594, University A)

Students noted that there are some positive messages of acceptance and civility being communicated over the internet, but that more attention should be given to building a positive culture online and on campuses. Two students from one focus group provided an example:

Josephine: Like for example the no make-up selfie campaign where somebody started it and encouraged women to not wear any make-up and take a picture of themselves and put it out there and knew the idea was to foster this, I guess, internet atmosphere of seeing people’s inner beauty […] like being counter-cultural on the internet and having a campaign that’s focused on beauty and value rather than on negative things is a good start.

Jane G.: And you can take that a step further with like you know like the hashtag stuff, do a hashtag campaign for it or kind of like what Dove does with their beauty campaign or something like that you know. […] or even the mental illness ones, right?

(University D, Focus Group 2)

Another focus group student lauded vloggers who routinely post warnings not to leave hate mail in the comments section as such statements would be removed (Blue, second-year, University B, Focus Group 2). One survey respondent mentioned their university’s Compliments page on Facebook and thought that worked well to promote a strong school community (Survey respondent 774, University A). Another survey respondent suggested a way to make the professor rating site more constructive: “Phrasing questions on sites like ratemyprofessors.com as not just general ‘comments’ where students are free to write malicious comments but in a way that asks for constructive criticism instead” (Survey respondent 856, University A).

Students also said that it was important to be proactive in thinking about how an online comment might be negatively perceived by the recipient, and to call out bad behaviour when one sees hurtful comments being made. “It could just be one word or something that didn’t mean to be cruel, but you just don’t know how other people are gonna take it” (Justin, second-year, University D, Focus Group 3). Or, as noted by another student: “There’s no sarcasm font” (Leelee, second-year, University A, Focus Group 2). A survey respondent wrote: “If you see cyber bullying, just speak up and let them know it’s uncool to be saying those things. Oftentimes they’re only bullying as a joke and don’t realise it’s hurtful” (Survey respondent 965, University A).

Students were also clear that universities need to be more proactive about creating a more positive campus culture overall, which would help to prevent and curtail cyberbullying. For instance,

Organize groups similar to fast friends that promote diverse friendships across faculties to reduce stereotypes and show commonalities among different ethnicities, genders, etc.

(Survey respondent 874, University A)

Students at University B, in particular, spoke about feeling disconnected from one another and that there was a lack of school spirit. University B has several campuses located in different regions and students felt as though there were few opportunities to get to know other students outside their own classroom or program. They felt that more opportunities to interact would increase one’s sense of belonging and community. As one student said: “like there are groups of friends, but I don’t know anyone at [university] *and I don’t think anyone knows me*” (Cher, first-year, University B, Focus Group 1—emphasis added). This feeling of isolation was not unique to University B. Some participants at University D also mentioned feelings of alienation:

I think what’s tough though is for the students who don’t have support groups or like really connections on campus to turn to and they just feel victimized from every corner of this building. […] *University can be very isolating*.

(Cherry Blossom, third-year, University D, Focus group 1—emphasis added).

Overall, students at all four universities said that their post-secondary experience and personal well-being would improve if universities took deliberate proactive measures to build a sense of community and belongingness on campus.

### 3.7. Disciplinary Measures

Implementing various disciplinary measures was among the top survey responses, although they were not emphasized in the focus group discussions. Some survey respondents felt that strong disciplinary measures, including public shaming, suspension and expulsion from university, should be part of the university’s response governing cyberbullying.

When someone is caught doing this to a student or faculty member, expose the bully to the entire university the way they did to their victim […]. Exposing and embarrassing the bully would be a deterrent for others, and give the bully “a taste of their own medicine” for lack of a better phrase. It’s not politically correct, but why do they deserve politically correctness? Some people would argue that embarrassing the bully might make them angry (or angrier) and cause them to lash out further, but I think it would be worth giving it a shot […]. Real discipline is the way it should be handled, and not privately behind closed doors, but open for all to see so everyone knows something is truly being done about it and that it is NOT TOLERATED.

(Survey respondent 58, University C)

Documenting proof and giving harsh punishments. Bullies should be held accountable for cyber bullying exactly like physical or verbal bullying.

(Survey respondent 198, University B)

Well in my previous example [multiple racist rants posted on a class site], the university could have done something. If they did do something, nobody in the class knows what it is... there was no apology and she kept coming to class. I think the girl should have been publicly reprimanded.

(Survey respondent 147, University A)

Although students generally believed that anonymity contributed in large measure to cyberbullying and should be reduced where possible, they did not take issue with anonymous reporting of cyberbullying. Survey respondents were strongly in favour of an anonymous reporting mechanism at the university. For example: “A system to report offenders would probably be helpful. The person reporting the cyber-bullying should be able to remain anonymous” (Survey respondent 208, University A).

Introduce stiff penalties that are enacted without exception. Introduce measures to help victims report abusers without having to do so publicly or in a confrontational setting. Implement a practice that would monitor for online abuses in common forums.

(Survey respondent 250, University A)

## 4. Conclusions

Students across all four Canadian universities identified several practical solutions to cyberbullying on their campuses. First and foremost, they felt that there was a general lack of awareness of the extent, nature, impacts, and consequences of cyberbullying at post-secondary. They would like to see the topic incorporated into orientation sessions at the start of each school year, and then reinforced each term and in each course, similar to how the problem of academic integrity is addressed (see also [21]). Students felt that university administrators did not take the problem of cyberbullying seriously enough, as reflected in the absence of awareness campaigns and the lack of clearly articulated policies and practices. Developing specific policies with stakeholders (including students) and communicating those policies was considered central to curtailing the problem. Policy development and clear communication of these policies and how to access them was the second strongest recommendation made by students—a recommendation that is consistently reinforced in the international research literature on this issue [1,3,11,19,43,44,45,48]. Students also felt that if universities gave greater priority to education and to policies regarding cyberbullying, together these actions would help to create an anti-cyberbullying culture, where negative online behaviour was frowned upon. It was interesting to us that students responding to the closed-ended survey question about solutions did not choose to rank education in their top five choices, although they did rank policy development as number five and developing a more respectful university culture as number three. These differences point to the value of including open-ended opportunities in surveys where students can write freely, as opposed to only providing pre-determined options posed by survey designers [64,65].

Students expressed mixed messages about the role and value of anonymity in online behaviour. On one hand, they appreciated the privacy that comes with being anonymous on the Internet, but they also recognized that anonymity can be a source of cyberbullying since it allows people to post whatever they wish online almost with impunity. This theme has been addressed in the research literature as well (see, for example, [6,21,38,39]). While students argued that more protections should be put in place to protect one’s privacy online (by self, by technology providers), several also recommended more invasive (less anonymous) ways to identify users, particularly on university platforms, including course sites. They also wanted to see mechanisms for anonymous reporting of abusers or targets, a recommendation also prioritized as number two by students in the closed-ended question on the survey. So, while survey and focus group respondents saw a role for university administrators and technology providers to reduce anonymity and provide greater protection for students, they were also worried about losing their own access to privacy. The students’ recommendations regarding anonymity were therefore not clear cut; rather, they revealed ironies and potential contradictions.

Some students, particularly survey respondents (less so from focus groups), felt that their peers should take greater responsibility to protect themselves in the online world, develop a tougher skin, and/or ignore the problem if they were targeted (a theme also taken up by Evans et al. [35]). A few students also placed blame on the victim for not taking sufficient precautions online, or for being too sensitive and over-reacting if targeted. This tendency to place blame on the victim has also been noted by other researchers (see [62,63]).

Both sets of respondents, however, noted the importance of bystander intervention in providing support to those targeted and in intervening if they see bullying happening. Students responding to the open- and closed-ended questions on the survey, and in the focus groups, drew attention to the importance of university stakeholders working together to build a greater sense of community and belonging on campus, even if the wider societal culture was not always a good role model.

In conclusion, it should be observed that the students in this study did not differ markedly in their recommendations regarding solutions in comparison with other post-secondary stakeholders. Dialogue with other stakeholders in post-secondary education (staff, faculty, administrators) revealed that they held beliefs similar to those of students about how to address the problem of cyberbullying in their institutions [57]. This compatibility of perspectives allows us to believe that the next steps are not a mystery. Moving forward requires recognition of the solutions that students and other stakeholders have raised and a willingness to take action.

Given that the issues with cyberbullying raised by this study in the Canadian context are similar to the problems identified in other jurisdictions (see [2]), we share these findings with the expectation that they have merit in other settings, while also encouraging universities elsewhere to undertake similar research with students so that their voices and opinions are heard. We note the value Chandler, Anstey and Ross [9] place on qualitative research to effect change.

The applicability and impact of qualitative research within the scientific community and in a greater social context is dependent on effective dissemination of the findings. Knowledge sharing is an integral component of the research process; without it, the potentiality of the research findings *to effect change* or benefit others is less probable.

(p. 1—emphasis added)

It is the hope of the present authors that this article will assist in effecting change regarding how solutions to cyberbullying in post-secondary institutions are developed and enacted.

## Appendix A. Focus Group Semi-Structured Questions

1. To what extent is cyberbullying a problem at university?
2. Why do you think it is occurring?
3. Who do you think mainly does the cyberbullying?
4. Who do you think are the primary victims?
5. What are some ways to prevent cyberbullying from occurring in the first place?
6. What are some ways to stop or curtail cyberbullying?
7. Why do you think these solutions might be effective?
8. What role does modeling play?
9. What role does dialogue and discussion play?
10. If you had the power to make one change at the university that would prevent, curtail or stop cyberbullying, what would that be?
11. What else would you like to tell us about the solutions to cyberbullying?

## Appendix B. Focus Group Participant Information

**Table A1:** Focus Group Participant Information

Pseudonym	Gender	University	Year of Study	Program
Leelee	Female	A	2	Criminology
Trieste	Female	A	3	Psychology
Flora	Female	A	3	Criminology
Yushi	Female	A	1	Undecided
Lux	Female	A	3–4	Chemistry/kinesiology
Han	Male	A	2	Geoscience
Kyle	Male	A	1	Business
Natalie	Female	A	4	Criminology
Julia	Female	A	4	Criminology
May	Female	A	4	Arts and Social Science
K	Female	A	1	Health Sciences
X	Female	A	4	Health Sciences
Berry	Female	B	2	Sociology
Cindy	Female	B	2	Psychology
Jay	Female	B	1	Psychology
Tim	Female	B	1	Health care studies
A	Female	B	3	Criminology
Christy	Female	B	1	Criminology/Psychology
Elena	Female	B	2	^1^
Cher	Female	B	1	Arts—general
DarkR0ze	Female	B	1	General studies
Blue	Male	B	2	Psychology
Flower	Female	B	3	Fashion design and technology
007	Male	B	1	Accounting
Saysha		C	2	Chemical engineering
Corvy		C	3	Arts—English/History
Hannah	Female	D	4	Honours Economics
Cherry Blossom	Female	D	3	Honours Economics
Giraldo	Male	D	4	Economics
Wallace	Male	D	1	Engineering
Jane G.	Female	D	4	Social work
Josephine	Female	D	3	English
Justin	Male	D	2	Physical Education
Sarah	Female	D	2	Business
Jan	Female	D	4	Chemistry
Emily	Female	D	4	Political Science

^1^ Spaces left blank indicate that no information was provided.
